# Supplementary material for: Landslide hazard cascades can trigger earthquakes
Source: Nat Commun. 2024 Apr 8;15:2878. doi: 10.1038/s41467-024-47130-w (PMC11001977; doi:10.1038/s41467-024-47130-w)
Supplement: Supplementary file 1 — Supplementary Information [file 41467_2024_47130_MOESM1_ESM.pdf]

Landslide hazard cascades can trigger earthquakes

Zhen Zhang[1]*, Min Liu[1], Yen Joe Tan[1]*, Fabian Walter[2], Siming He[3], Małgorzata Chmiel[4,2], Jinrong Su[5]

[1]Earth and Environmental Sciences Programme, Faculty of Science, The Chinese University of Hong Kong, Hong Kong S.A.R., China

[2]Swiss Federal Institute for Forest, Snow and Landscape Research, Zürich, Switzerland

[3]State Key Laboratory of Mountain Hazards and Engineering Safety, Institute of Mountain Hazards and Environment, Chinese Academy of Sciences, Chengdu, China

[4]Géoazur, OCA, Campus Azur du CNRS, Sophia Antipolis, France

[5]Earthquake Monitoring Center, Sichuan Earthquake Administration, Chengdu, China

*Corresponding author. Email: zhenzhang@cuhk.edu.hk (Z.Z.); yjtan@cuhk.edu.hk (Y.J.T.)

Contents of this file

Figures S1 to S18
Table S1

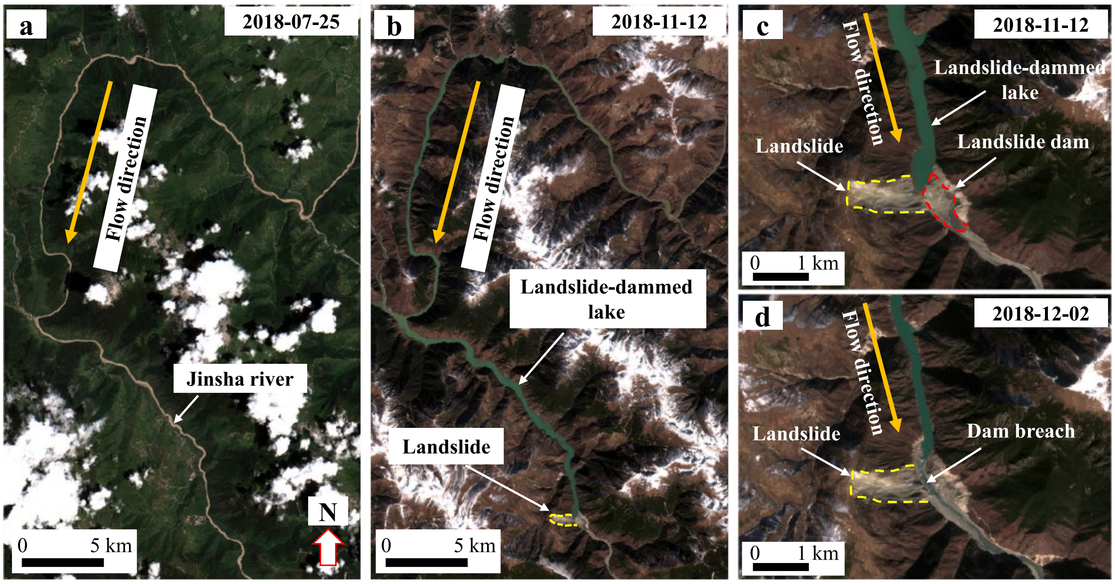

Fig. S1 | Sentinel-2 images covering the 2018 Baige landslides (source: the Copernicus Data Space Ecosystem). Dashed yellow and red lines outline the landslides and dams in b, c, and d. The times of these images are displayed in yyyy-mm-dd format in each panel.

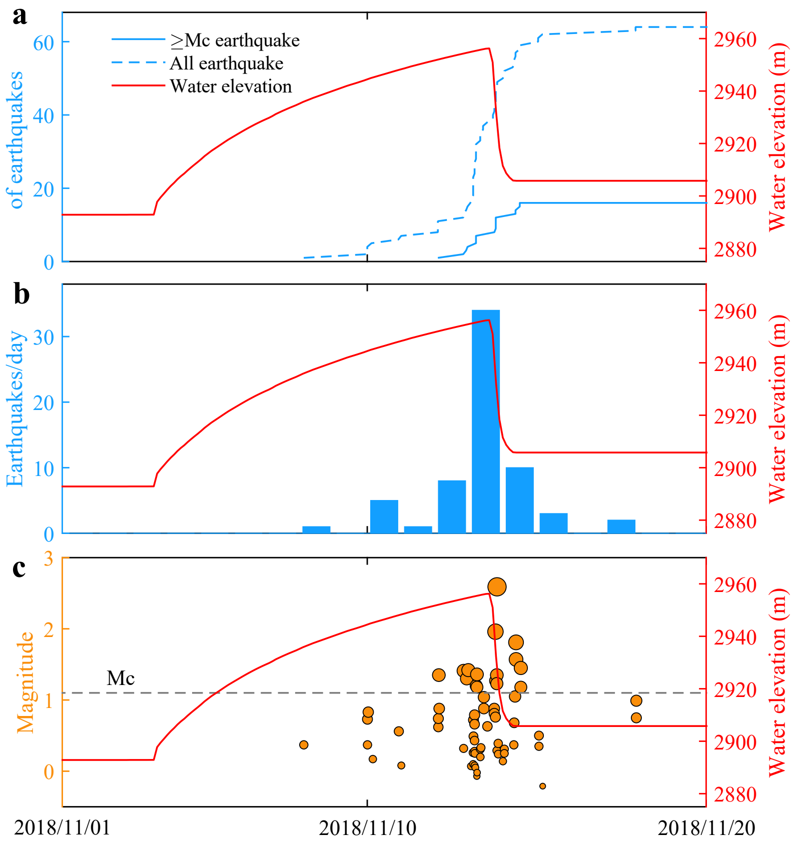

Fig. S2 | Cumulative number of earthquakes, daily seismicity rate, earthquake magnitudes, and landslide-dammed lake water level over a twenty-day period. Gray dashed line in (c) marks the magnitude of completeness.

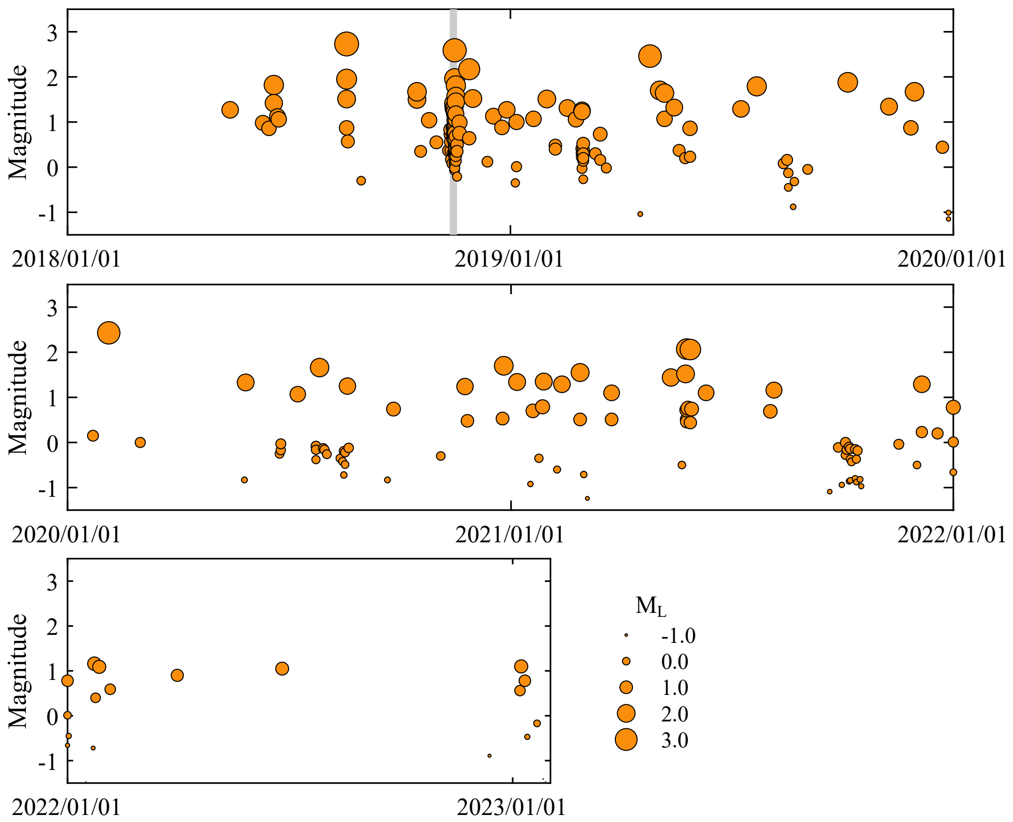

Fig. S3 | Local magnitude of earthquakes within 10 km of the Baige landslide-dammed lakes (LDLs) over a five-year period. Gray bar marks the week (from 10 to 16 November, 2018) when the second LDL approached its peak water level.

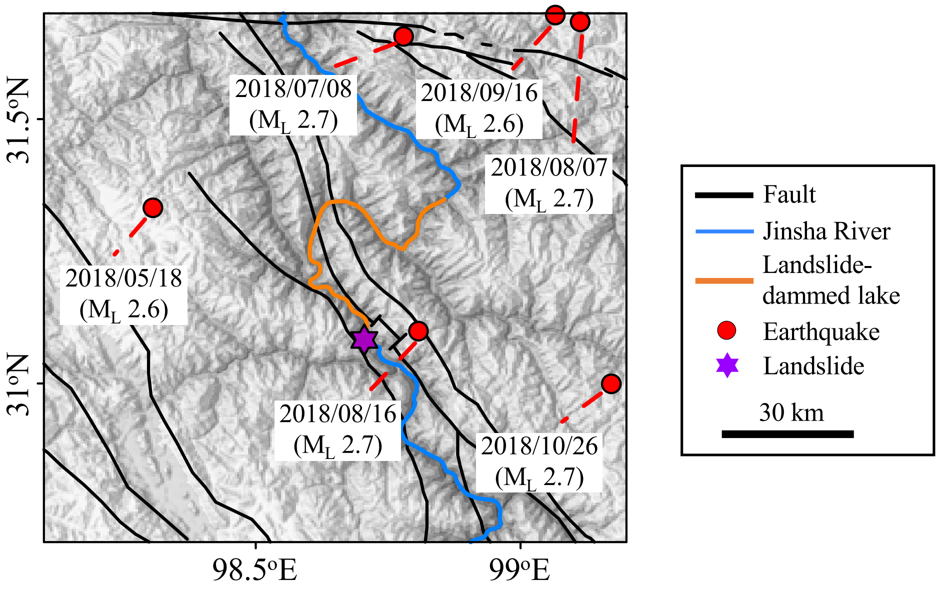

Fig. S4 | Distribution of M_L>2.5 earthquakes near the Baige landslides from May 2018 to November 2018. Times and magnitudes of the earthquakes are indicated.

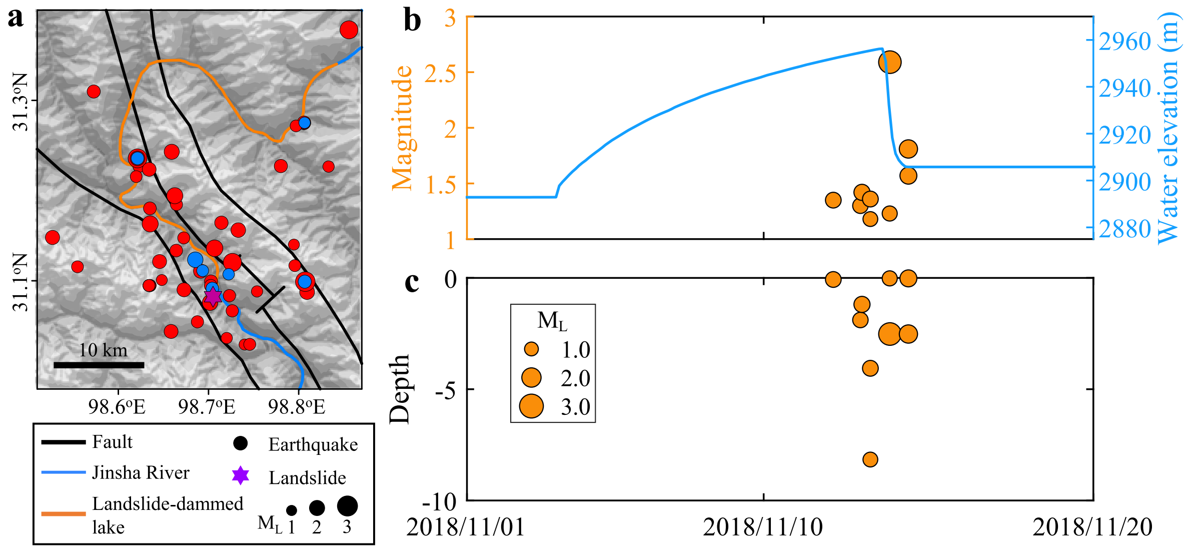

Fig. S5 | Earthquakes after performing declustering. a Distribution of earthquakes from May 2018 to January 2023 within 10 km from landslide-dammed lakes (LDLs). Red and blue dots represent the locations of earthquakes that were left and removed after performing declustering, respectively. **b** Earthquake magnitudes and LDL water level over a twenty-day period. **c** Earthquake depths over a twenty-day period.

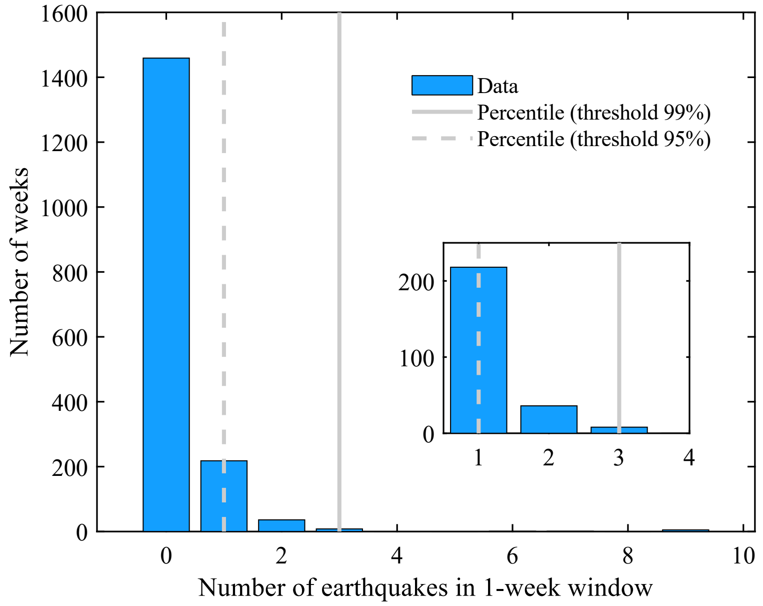

Fig. S6 | Number of earthquakes in a given 1-week time window using the earthquakes from May 2018 to January 2023. The inset figure zooms into a region of the histogram with smaller values.

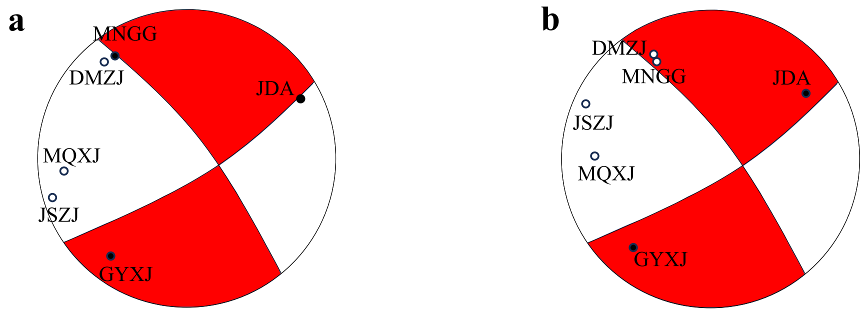

Fig. S7 | Focal mechanism of M_L 3.7 earthquake and the first motions of two M_L>1.8 earthquakes at their surrounding seismic stations. Red beach balls depict focal mechanisms of the M_L 3.7 earthquake that occurred on 30 June 2023 (striking: 323°, dip: 74°, slip: -16°) in Fig. 1b. For the earthquake sequence which occurred within 10 km of the landslide-dammed lake (LDL) as the second LDL approached peak water level, the first motions of the two largest earthquakes (M_L 2.6 (**a**) and M_L 1.9 (**b**)) at surrounding seismic stations are marked. Black and white dots represent upward and downward first motions, respectively.

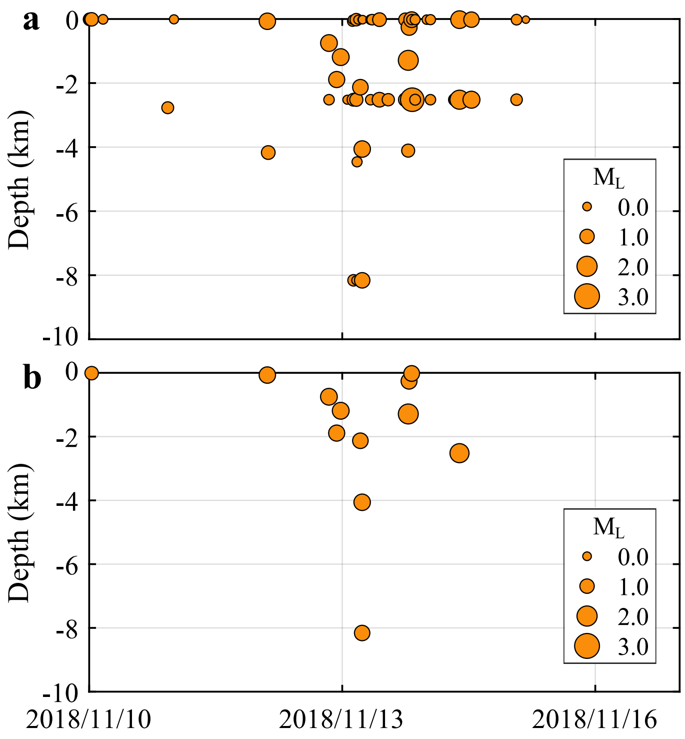

Fig. S8 | Earthquake depths in complete catalog (a) and in catalog before template matching (b) over an eight-day period. Median earthquake depths in complete catalog and in catalog before template matching are 1.19 and 1.24 km, respectively.

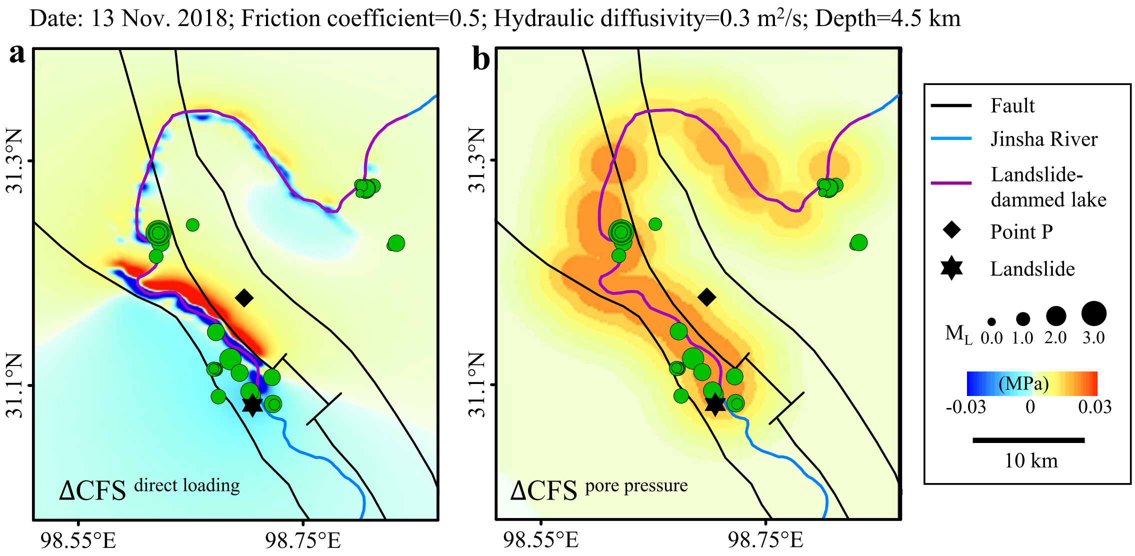

Fig. S9 | Coulomb stress change (ΔCFS) map on 13 Nov 2018. ΔCFS on the surrounding faults due to the direct gravitational loading (**a**) and pore pressure diffusion (**b**).

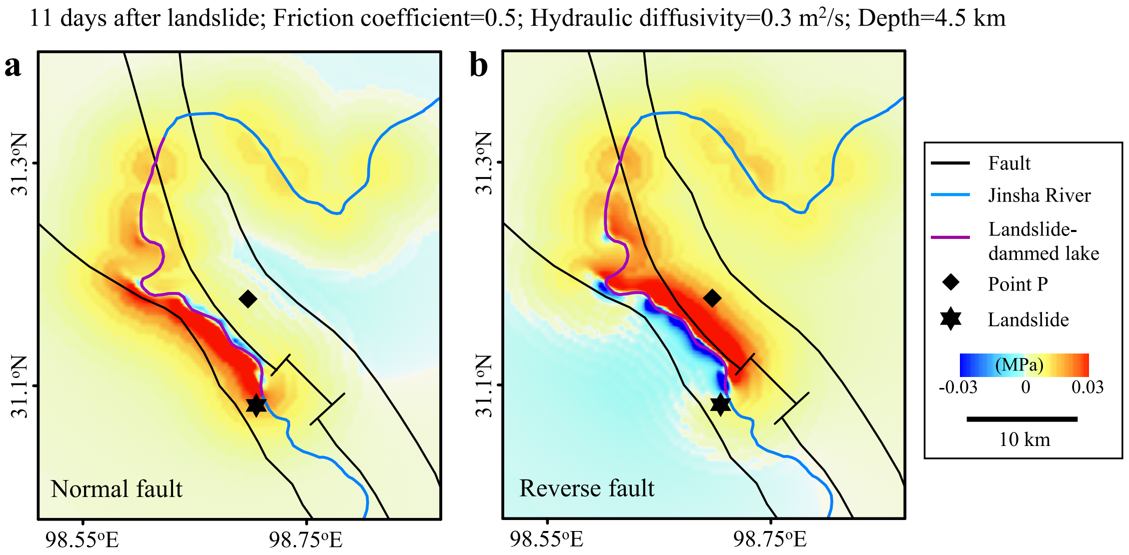

Fig. S10 | Coulomb stress change (ΔCFS) map at depth of 4.5 km corresponding to -0.5 km asl, 11 days after the formation of a 30-m deep landslide-dammed lake (LDL). ΔCFS on the surrounding normal (**a**) and reverse (**b**) fault systems due to the LDL's direct gravitational loading and pore pressure diffusion.

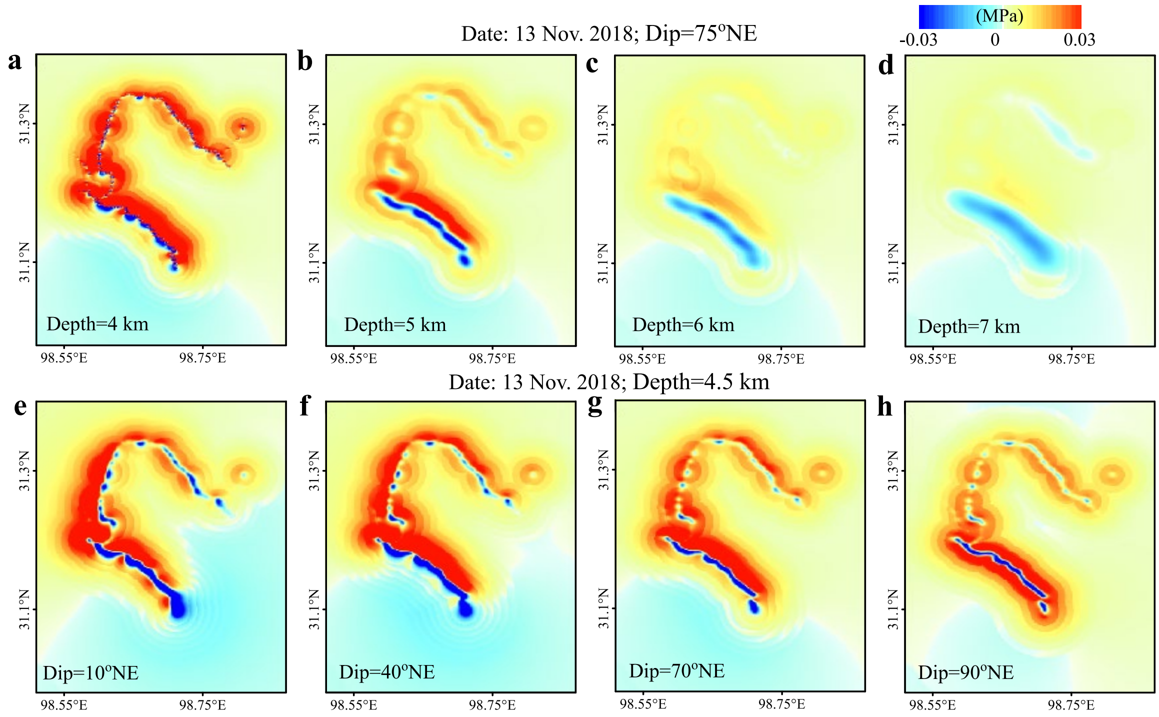

Fig. S11 | Coulomb stress change (ΔCFS) map on 13 Nov 2018. a-d ΔCFS, as in Figure 2, assuming different fault depths. **e-h** ΔCFS assuming different fault dip values.

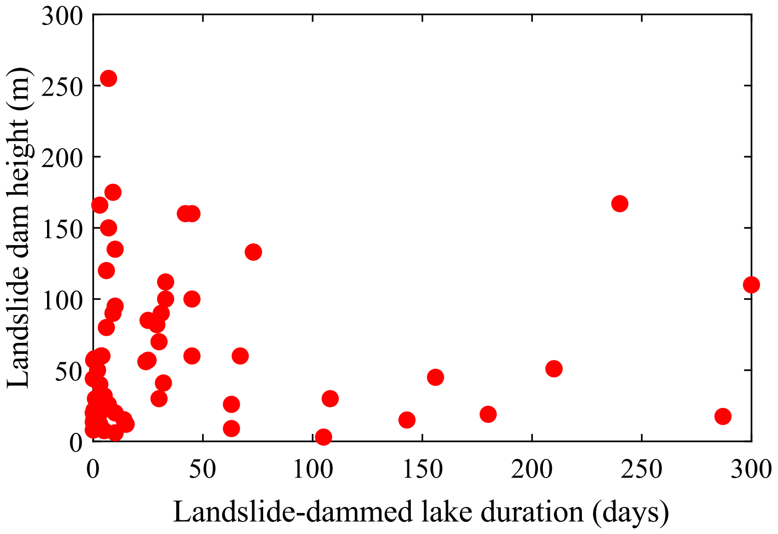

Fig. S12 | Landslide dam height versus landslide-dammed lake duration[50].

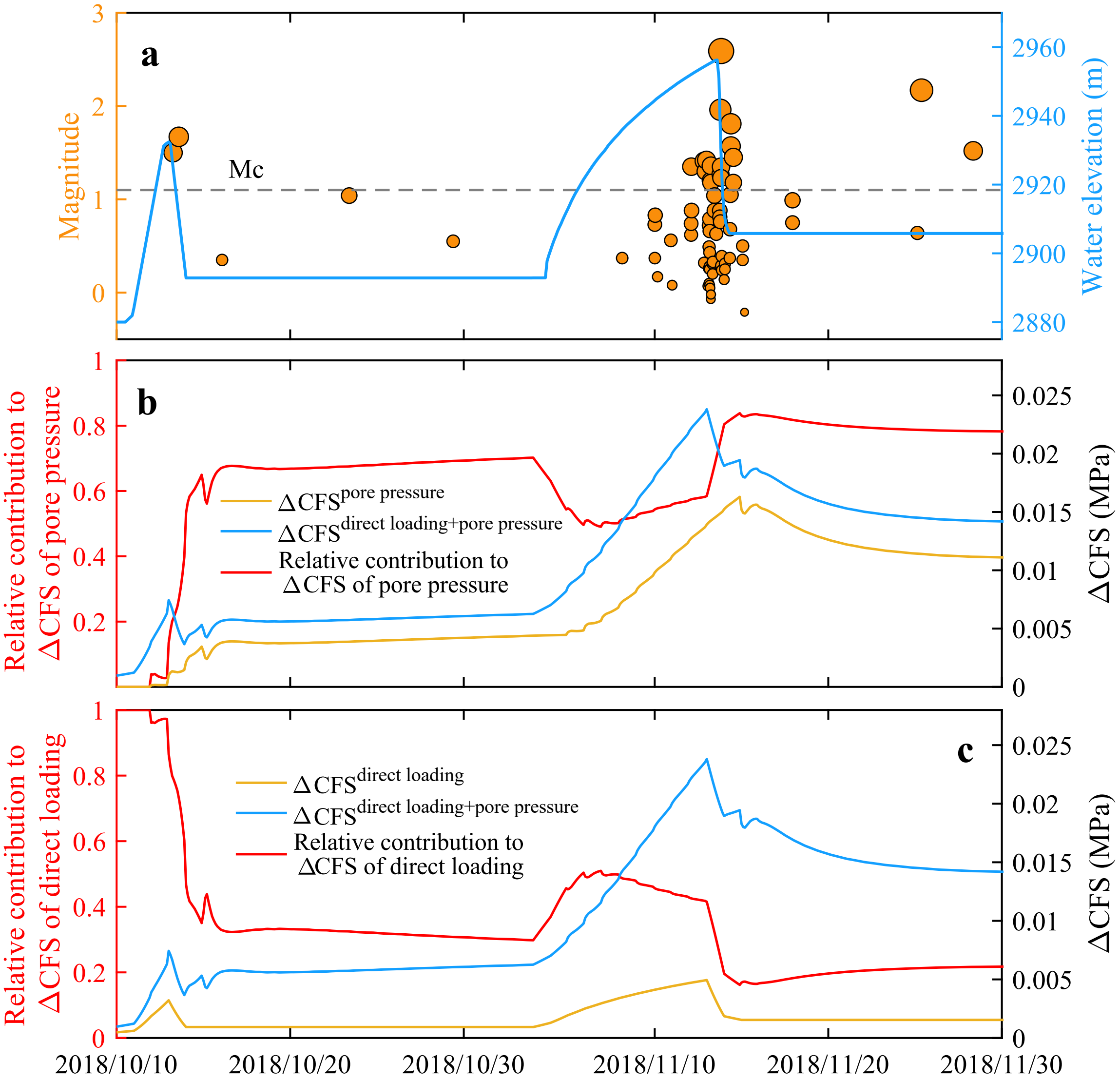

Fig. S13 | Relative contributions to Coulomb stress change (ΔCFS) of pore pressure and direct loading and earthquakes triggered by the 2018 Baige landslide-dammed lake (LDL). a Earthquake magnitudes and LDL water level over a fifty-day period. Gray dashed line marks the magnitude of completeness Mc. Relative contributions to ΔCFS of pore pressure (**b**) and direct loading (**c**) at point P (Fig.2a).

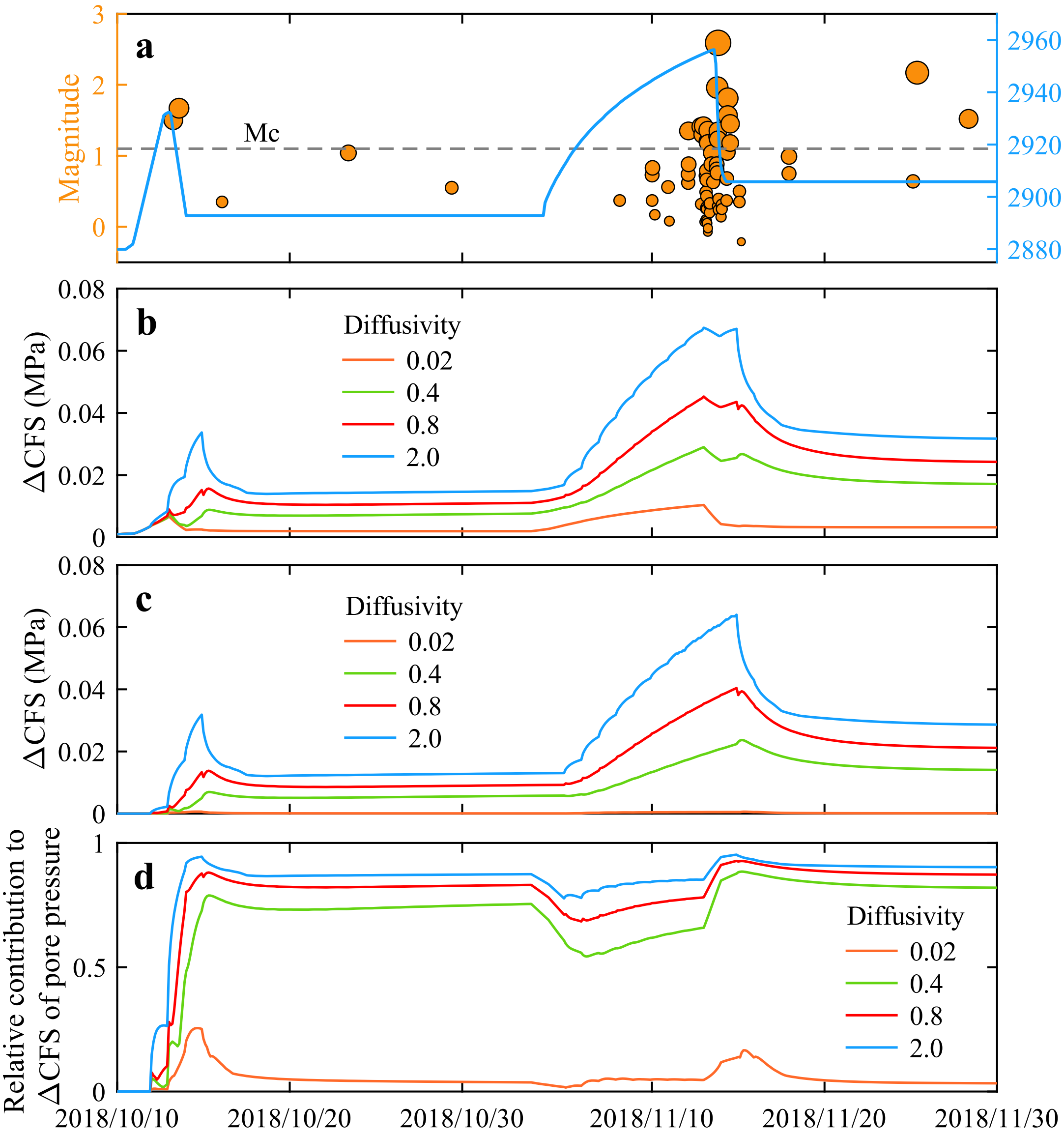

Fig. S14 | Coulomb stress change (ΔCFS) and earthquakes triggered by the 2018 Baige landslide-dammed lakes (LDLs). a Earthquake magnitudes and LDL water level over a fifty-day period. Gray dashed line marks the magnitude of completeness Mc. **b** Temporal evolution of ΔCFS due to the combined effect of direct gravitational loading and pore pressure diffusion at point P (Fig. 2a) assuming different values of hydraulic diffusivity coefficient. **c** Temporal evolution of ΔCFS due to pore pressure diffusion at point P (Fig. 2a) assuming different values of hydraulic diffusivity coefficient. **d** Relative contribution to ΔCFS of pore pressure at point P (Fig.2a) assuming different values of hydraulic diffusivity coefficient.

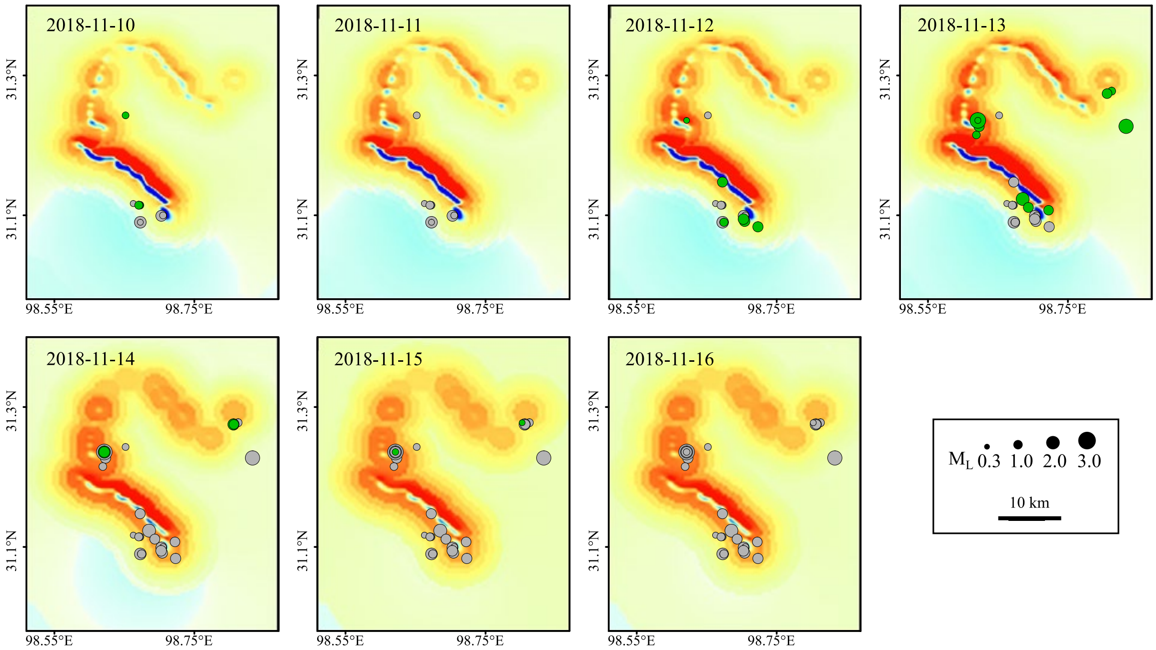

Fig. S15 | Coulomb stress change (ΔCFS) maps at depth of 4.5 km and earthquakes that occurred before (grey filled circles) and on (green filled circles) each day from 10 to 16 Nov 2018.

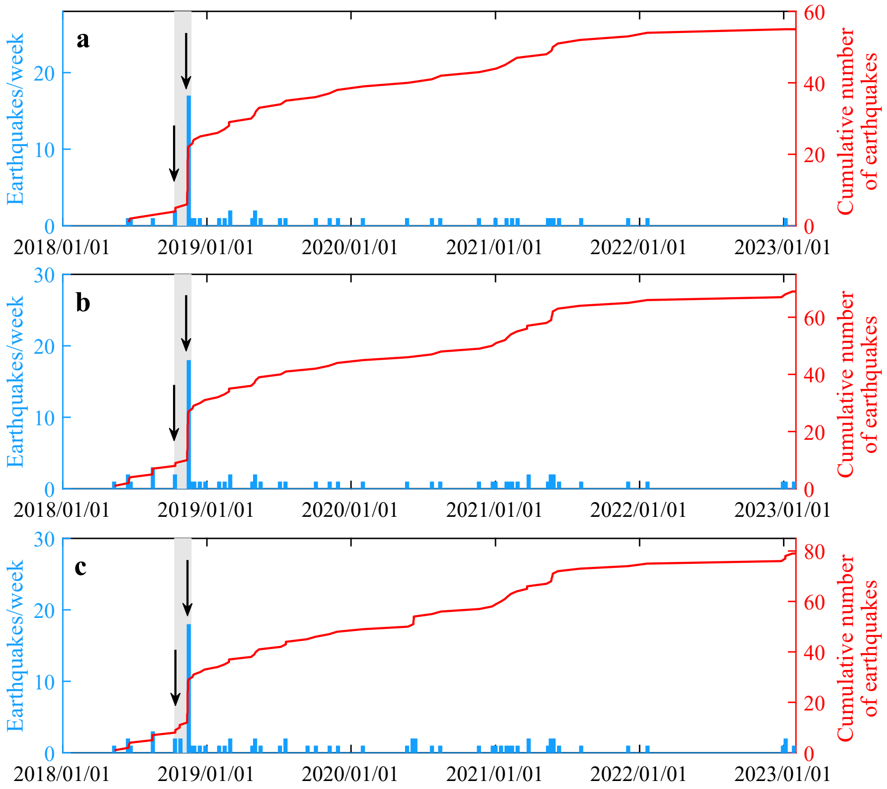

Fig. S16 | Cumulative numbers of earthquakes with local magnitudes greater than or equal to the magnitude of completeness (Mc =1.1) which occurring within 8 km (a), 12 km (b), and 14 km (c) of the landslide-dammed lakes over a five-year period. Arrows mark the timings of the two 2018 Baige landslides.

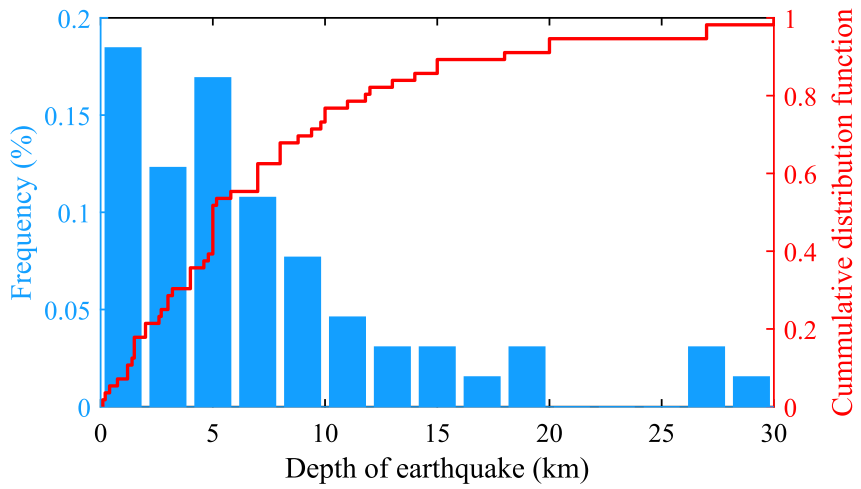

Fig. S17 | Frequency distribution and cumulative distribution function of the maximum depth of reservoir-induced earthquakes[49].

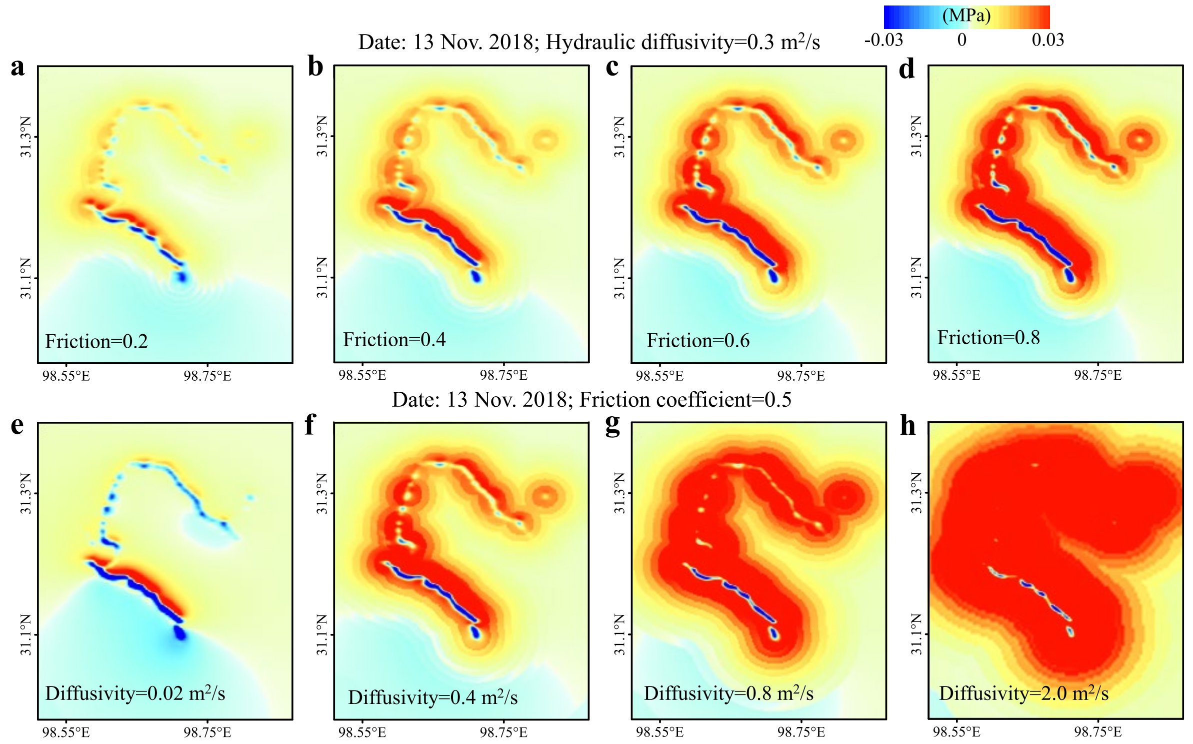

Fig. S18 | Coulomb stress change (ΔCFS) on 13 November 2018. a-d ΔCFS, as in Figure 2, assuming different values of friction coefficient. **e-h** ΔCFS assuming different values of hydraulic diffusivity coefficient.

Table S1 | Seismicity rate change and statistical significance.

Time window	Seismicity rate per week		
	≥Mc events	Declustered events with input I**	Declustered events with input II***
1 week during the second LDL*	16	9	5
27 weeks before the second LDL (Total number of earthquakes)	0.3 (9)	0.3 (8)	0.3 (8)
224 weeks after the second LDL (Total number of earthquakes)	0.2 (37)	0.2 (37)	0.2 (37)
Maximum over observation period besides the second LDL	3	3	3
Statistical tests	Statistical significance		
	≥Mc events	Declustered events	Declustered events
Statistic *P*	100.00%	99.82%	97.41%
Statistic *Z*	100.00%	99.62%	96.52%
Improved statistic based on Poisson probability	100.00%	99.73%	89.92%
Empirically derived statistic	99.71%	99.71%	99.71%

* 1 week (10 to 16 November, 2018) when the second landslide-dammed lake (LDL) approached its peak water level

** look-ahead time from 0.5 to 3 days, confidence probability of 0.9, effective min magnitude cutoff of 1.1, the increase in lower cutoff magnitude during clusters of 0, and the number of crack radii surrounding each earthquake of 5

*** look-ahead time from 1 to 10 days, confidence probability of 0.95, effective min magnitude cutoff of 1.5, the increase in lower cutoff magnitude during clusters of 0.5, and the number of crack radii surrounding each earthquake of 10
